# In Vitro Anti-Inflammatory Activities of Fucoidans from Five Species of Brown Seaweeds

**DOI:** 10.3390/md20100606

**Published:** 2022-09-27

**Authors:** Ekaterina D. Obluchinskaya, Olga N. Pozharitskaya, Alexander N. Shikov

**Affiliations:** 1 Murmansk Marine Biological Institute of the Russian Academy of Sciences (MMBI RAS), 17 Vladimirskaya str., Murmansk 183010, Russia; 2 Department of Technology of Pharmaceutical Formulations, St. Petersburg State Chemical Pharmaceutical University, 14a Prof. Popov str., Saint Petersburg 197376, Russia

**Keywords:** anti-inflammatory, fucoidan, isolation, antioxidant, polyphenols, brown algae, synergy, protein denaturation

## Abstract

This study aimed to compare the anti-inflammatory effects of fucoidans from brown seaweeds (*Saccharina japonica* (SJ), *Fucus vesiculosus* (FV), *Fucus distichus* (FD), *Fucus serratus* (FS), and *Ascophyllum nodosum* (AN)), and determine the relationship between composition and biological activity. The anti-inflammatory activity was tested in vitro. It is believed that inflammation could be triggered by free radicals. Fucoidans from *F. vesiculosus* (FV1 and FV3) showed the strongest 1,1-diphenyl-2-picrylhydrazyl (DPPH) radical scavenging activity with an IC_50_ = 0.05 mg/mL. In the total antioxidant capacity (TAC) test, the activity was concentration-dependent. Notable, the TAC of fucoidans except samples of FV2 and SJ (which have a lower phenolic content) was higher than that of phloroglucinol. The TAC of fucoidans strongly and positively correlated with polyphenol content. A weak correlation was associated with xylose content. The synergistic effect for fucoidans was calculated for the first time using carbohydrates and polyphenols as model mixtures. The synergy in the DPPH test was found only for FV1 and FV3 (mixture effect ME = 2.68 and 2.04, respectively). The ME strongly positively correlated with polyphenols. The relationship of ME with fucose content was positive but moderate. It was first established that the anti-inflammatory effects of fucoidan could be mediated via the inhibition of protein denaturation. The inhibition was concentration-dependent and strongly correlated with the fucose content and moderate with sulfate content. The purified fucoidan FV2 showed the most promising activity (IC_50 =_ 0.20 mg/mL vs. IC_50 =_ 0.37 mg/mL for diclofenac sodium). Similar relations were also observed in the membrane protection model. Fucoidans were able to stabilize the cell membrane integrity of human red blood corpuscles (HRBC). The results of our study support the rationality of fucoidan use as a promising agent for the treatment of inflammatory-related diseases via mechanisms of radical scavenging, antioxidant activity, inhibition of protein denaturation, and HRBC membrane stabilization.

## 1. Introduction

Brown seaweeds are a rich source of anti-inflammatory compounds with diverse combinations of chemical variations. Such biologically active principles range from polyphenols, polysaccharides, fatty acids, and halogenated compounds to carotenoids [1]. Among them is sulfated polysaccharide fucoidan, which contains 20–60% fucose with alternating α-glycosidic bonds, and dominates as a potent and widely studied biomolecule [1,2,3]. Fucoidan from brown seaweeds essentially contains fucose and sulfate groups along with other sugars such as galactose, xylose, and mannose [4,5]. Fucoidan is an increasingly recognized bioactive substance used in cosmetics, functional foods, nutritional supplements, animal feed, and pharmaceuticals, including incorporating fucoidan nanocomposites into a three-dimensional framework [6,7]. However, the structure of fucoidan is variable and depends on the species and harvesting season of seaweeds, the changes in the content and sulfation of monosaccharides, and different extraction methods [6,8,9,10]. All this affects the commercialization of fucoidan-based preparations. Therefore, bioactive fucoidan must be isolated from different species of brown seaweeds and tested. This ensures the quality of fucoidan with unambiguous pharmaceutical effects, which further allows the standardized preparation to be sold on the commercial market.

Inflammation is a complex reaction of the organism. Free radicals are recognized as mediators of inflammation. In addition to promoting direct toxicity, reactive oxygen metabolites may also initiate and/or amplify inflammation [11]. Inflammation is accompanied by an imbalance of the immune system, an increase in vascular permeability, protein denaturation, etc. [12]. Protein denaturation induces the production of autoantigens in such conditions of inflammation as cancer and diabetes. Production of autoantigens in certain arthritic diseases may be due to the denaturation of proteins [13]. The inflammatory activity can be suppressed by inhibiting protein denaturation [14]. Although several anti-inflammatory mechanisms of fucoidan were reported [15,16,17,18], very little is known about the ability of fucoidans to prevent protein denaturation. Agents that can prevent protein denaturation would be worthwhile for anti-inflammatory drug development.

Following our previous results [15,19], in this study, we aimed to compare the anti-inflammatory effects of fucoidans from five brown seaweeds (*Saccharina japonica*, *Fucus vesiculosus*, *Fucus distichus*, *Fucus serratus*, and *Ascophyllum nodosum*), and determine the relationship between composition and biological activity.

## 2. Results and Discussion

### 2.1. Fucoidan Compositions

**Figure 1:**
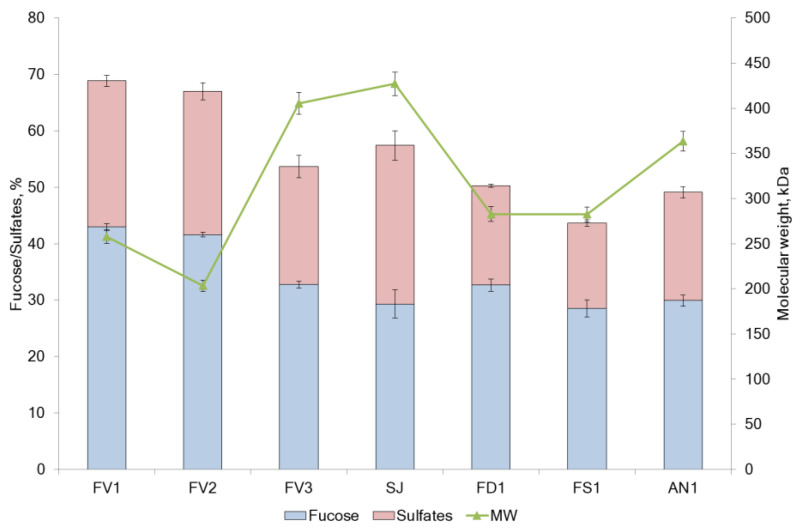
Composition of fucoidans from the brown algae. *F. vesiculosus* (FV1, FV2, FV3), *S. japonica* (SJ), *F. distichus* (FD1), *F. serratus* (FS1), and *A. nodosum* (AN1).

Several brown seaweed species were extracted to investigate their potential bioactivities. The chemical compositions of fucoidans are summarized in Table 1 and Figure 1.

The yield of fucoidans varied between 6.6 and 22.3% of the seaweed dry weight (DW). The main components were neutral carbohydrates (44.5–65.9 g/100 g DW) and sulfate (15.1–28.1 g/100 g DW). Samples FD1 and FS1 contained a high ratio of neutral carbohydrates to sulfates. SJ showed a high neutral carbohydrate content (Table 1) but the lowest fucose content (Figure 1). The yield of the additionally purified from phenolic compounds sulfated polysaccharide (FV2) represented 6.6% of DW of seaweeds, and its proportion of sulfate from neutral carbohydrate (2.45) almost coincided with the proportion of FV1 (2.54). Minor components in all samples were polyphenols with an average of 70.6 mg/g DW (Table 1).

**Figure 2:**
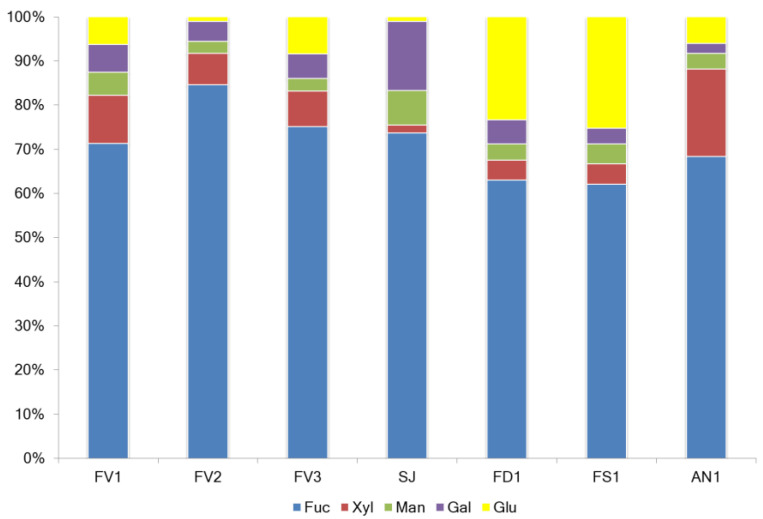
Normalized histogram of monosaccharide composition of fucoidans extracted from the brown algae. *F. vesiculosus* (FV1, FV2, FV3), *S. japonica* (SJ), *F. distichus* (FD1), *F. serratus* (FS1), and *A. nodosum* (AN1). Fucose (Fuc), xylose (Xyl), mannose (Man), galactose (Gal), and glucose (Glu).

The content of fucose in the samples ranged from 28.1% in SJ to 43.0% in FV1 and FV2, respectively (Figure 1). Analysis of the monosaccharide composition of the extracted fucoidans showed that it belongs to the F-type and contained mainly fucose from 62.1 mol.% in FS1 to 75.1 mol.% in FV1, respectively. The highest fucose value (84.6 mol.%) was found in sample FV2, additionally purified from polyphenols (Figure 2). Fucoidans obtained from *F. distichus* and *F. serratus* were enriched with glucose in the composition up to 23.3–25.3 mol.%, respectively. Thus, fucoidan extracted from *F. vesiculosus*, independent of the extraction method, belongs to fucans, while that from *F. distichus* and *F. serratus* are fucoglucans. Fucoidan extracted from *A. nodosum* is enriched in xylose 19.8 mol.% and belongs to fucoxylans. Commercial sample from *S. japonica* with a high percentage of galactose (15.7 mol.%) could be classified as fucogalactans [20].

Different methods (Method I [21], Method II [22] and Method III [23]) were used in this study for the extraction of fucoidan from *F. vesiculosus*. The yield of fucoidan FV1 isolated by Method I was 2.7 folds higher than the yield of fucoidan FV3 extracted by Method III (Table 1). The molecular weight of fucoidans was 257.8 kDa and 405.5 kDa for FV1 and FV3, respectively (Figure 1). Method I provides a much higher content of neutral carbohydrates (64.1 ± 0.6% in FV1) vs. 53.7 ± 0.6% in sample FV3 (Method III). A similar trend was observed for the sulfate content (25.9% in sample FV1 vs. 20.9% in sample FV3). These differences could be due to the application of ultrasound-assisted extraction followed by ultrafiltration to remove alginate by Method I. The total neutral carbohydrate and sulfate content decreased after treatment of seaweed with CaCl_2_, which was used in Method III to obtain fucoidan FV3.

The sample FV2 was obtained using Method II after purification of sample FV1. A hydrophobic resin Polychrome-1, recommended by [22], was used for the purification of FV1 from non-polar compounds (pigments, some proteins, polyphenols, etc.). The yield of FV2 was comparable with the yield of FV3, obtained by Method III (Table 1). Additional purification on Polychrome-1 did not lead to a significant increase in the content of fucose and sulfates compared to Method I (Figure 1). At the same time, a change in the monosaccharide composition of fucoidan was observed. The rate of fucose was increased, while other monosaccharides were decreased (Figure 2). The content of total polyphenols did not differ between the two extraction methods, while it was ten folds less in purified sample FV2. Taking together all these data, we believe that Method I is preferable for the extraction of fucoidan from *F. vesiculosus*.

### 2.2. Anti-Inflammatory Activity

The therapy of inflammation (which is considered to be a complex disease of the organism) requires multitarget agents with different mechanisms. Although some anti-inflammatory mechanisms have been described in the literature for fucoidan, it is reasonable to explore correlations between composition and activity. The activity was tested in vitro.

#### 2.2.1. The Antiradical and Total Antioxidant Activities

It is believed that inflammation could be triggered by free radicals. Free radicals produced by inflammatory cells cause degradation of DNA, affect cell proliferation, apoptosis, etc., which lead to carcinogenesis [24]. Reactive oxygen metabolites may also initiate and/or amplify inflammation [11]. The antiradical/antioxidant activities of fucoidan were evaluated using two in vitro test systems.

**Figure 3:**
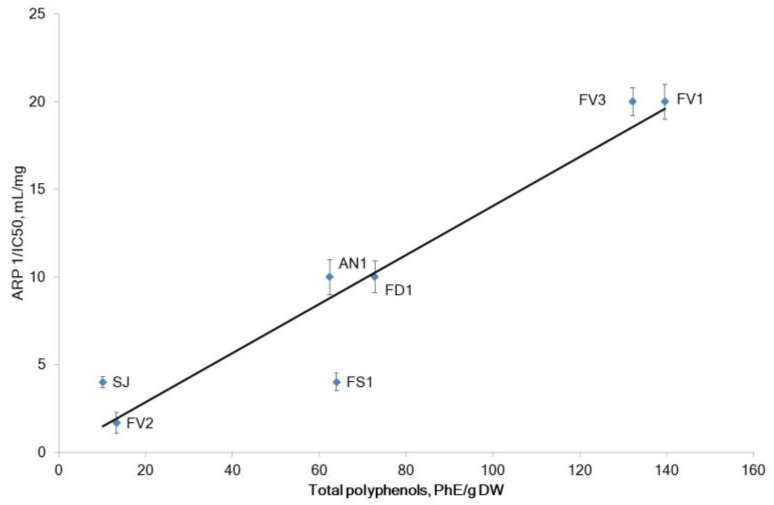
The antiradical power (ARP) and total polyphenols content (TPC) in (phloroglucinol equivalents/g DW for fucoidans extracted from the tested brown seaweeds. Values are expressed as the mean ± SD. *F. vesiculosus* (FV1, FV2, FV3), *S. japonica* (SJ), *F. distichus* (FD1), *F. serratus* (FS1), and *A. nodosum* (AN1).

Commonly, the radical scavenging potential of seaweed extracts is associated with phenolic compounds [25]. In our experiments, the antiradical power (ARP) (tested by the scavenging of DPPH-radicals) for each fucoidan sample and the total phenolic content (TPC) measured by the Folin–Ciocalteu method are shown in Figure 3. Fucoidan from *F. vesiculosus* (samples FV1 and FV3) showed the most prominent radical scavenging activity with an IC_50 =_ 0.05 mg/mL, probably due to its high polyphenol content of about 135.9 PhE/g DW. The other four seaweeds showed a relatively lower radical-scavenging level (IC_50 =_ 0.1–0.25 mg/mL). All these samples also contained lower levels of phenolic compounds (62.4–72.8 PhE/g DW). The purified fucoidan FV2 from *F. vesiculosus* FV1 had a weak ARP, apparently due to low polyphenolic content. The order of the ARP for the tested fucoidans was FV1 = FV3 > FD1 = AN1 > SJ = FS1 > FV2 (Figure 3).

A strong positive correlation of ARP and TPC (Pearson’s correlation coefficients r = 0.95, *p* < 0.05) was noted. Our results are consistent with previous studies that reported a direct correlation between DPPH scavenging activity and polyphenolic compounds for seaweed extracts [9,26,27]. We have observed a weak direct correlation between xylose level and ARP (Pearson’s correlation coefficient r = 0.35, *p* < 0.05), while the rate of other sugars was not correlated with ARP. The method of extraction (sample FV1 vs. FV3) showed no significant effect on the DPPH-radical scavenging activity by fucoidans.

**Figure 4:**
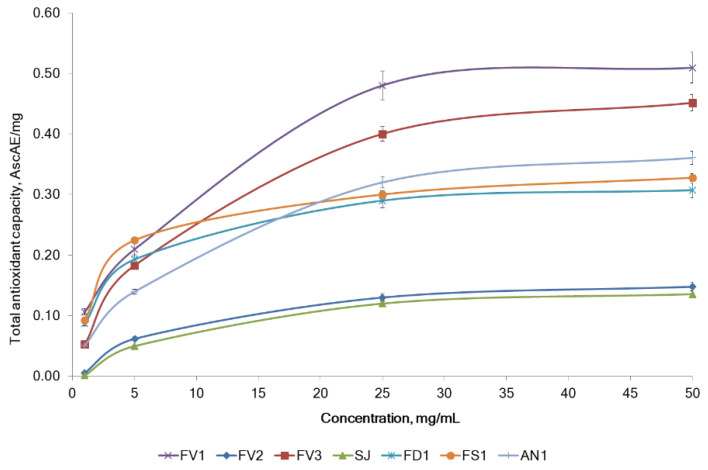
Total antioxidant capacity (TAC) of fucoidans extracted from the brown algae. *F. vesiculosus* (FV1, FV2, FV3), *S. japonica* (SJ1), *F. distichus* (FD1), *F. serratus* (FS1), and *A. nodosum* (AN1).

**Figure 5:**
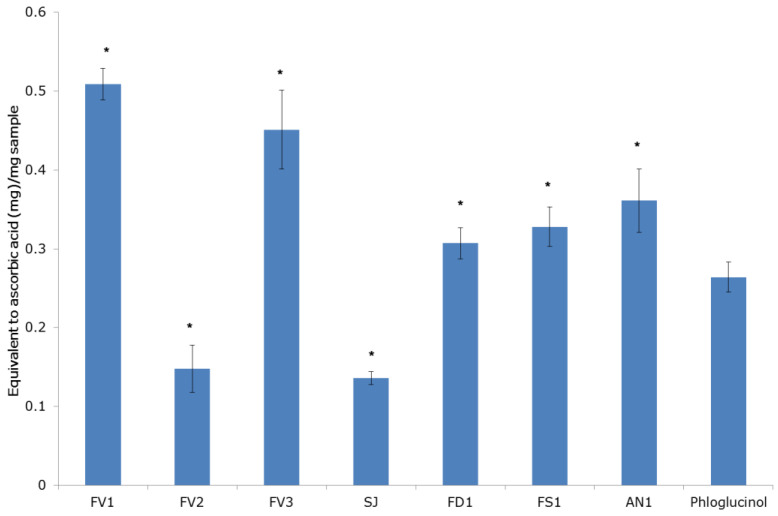
Total antioxidant capacity (TAC) of fucoidans at 50 mg/mL concentration, extracted from the brown seaweeds. *F. vesiculosus* (FV1, FV2, FV3), *S. japonica* (SJ), *F. distichus* (FD1), *F. serratus* (FS1), and *A. nodosum* (AN1). * A significant (*p* < 0.000) difference was found between the phloroglucinol group (reference drug) and fucoidan groups.

The analysis of TAC of fucoidans was based on the reduction of Mo(VI) to Mo(V) by the samples and the subsequent formation of a green phosphate/Mo(V) complex at acid pH. The TAC was quantitatively expressed in equivalents of ascorbic acid [28]. The tested fucoidans showed total antioxidant activity at different concentrations (1–50 mg/mL). The TAC of all fucoidan samples concentration-dependent increased up to 25 mg/mL, followed by a plateau up to 50 mg/mL (Figure 4). Fucoidans FV1 and FV3 from *F. vesiculosus* had a higher TAC than the other fucoidans. The maximum activity of 0.51 ± 0.03 mg AscAE/mg was observed for fucoidan FV1 at 50 mg/mL (Figure 5). The order of the TAC for all the tested seaweed fucoidans was FV1 > FV3 > AN1 > FS1 > FD1 > FV2 > SJ (Figure 5). Notable that the TAC of fucoidans except samples of FV2 and SJ (which have a lower polyphenolic content) was higher than that of phloroglucinol (Figure 5). In the TAC assay, the activities of fucoidans FV1 and FV3 extracted by different methods were equal.

According to L. Nurhidayati et al. [29], the higher sulfate content in a crude fucoidan from *Sargassum cinereum* was associated with a higher ability to reduce DPPH radicals. However, we did not find a correlation between sulfate content and DPPH scavenging activity by fucoidans from five tested seaweeds, while a low negative correlation (Pearson’s correlation coefficients r = −0.302, *p* < 0.05) between the TAC and sulfates content was observed. Other authors have suggested that the molar ratio of sulfate content to fucose could influence the antioxidant activity due to the reaction ability of not only the sulfate group but also the di-substitute fucose units with free radicals [30]. In the current study, the molar ratio of sulfate content to fucose was positively correlated with TAC (Pearson’s correlation coefficients r = 0.425, *p* < 0.05). Apart from the fucoidan and polyphenols, brown seaweed contains other secondary metabolites, which could be co-extracted and are believed to possess antioxidant activity [31].

Although two methods (DPPH radical scavenging activity and total antioxidant activity) were applied for the testing of fucoidan samples, the outcomes of different models were similar (Pearson’s correlation coefficients r = 0.80, *p* < 0.05).

#### 2.2.2. Determination of Mixture Effect

Seaweed fucoidans represent a complex mixture of compounds, which could contribute to their antiradical activity. Understanding the role of individual compounds/fractions in the antioxidant activity of natural extracts is challenging [32]. One of the methods for assessing synergistic or antagonistic effects of the combination of individual compounds/fractions is the calculation of a mixture effect (ME) [32,33]. Computationally, ME > 1 evidence a synergistic effect between antioxidants; in case ME < 1, an antagonism is observed; ME = 1 indicate the absence of synergistic or antagonistic effects.

Although several authors associate a high antioxidant activity of extracts from brown seaweeds with a synergy of extract components [34,35], the contribution of individual active compounds in synergy was not supported by mathematical calculations. Recently, we have calculated ME for EtOH and natural deep eutectic solvent (NADES) extracts from *F. vesiculosus*. The high synergy in the DPPH scavenging test by EtOH and NADES extracts (ME = 2.03 and ME = 2.27, respectively) was explained mainly by the contribution of ascorbic acid and phenolic compounds [19].

**Figure 6:**
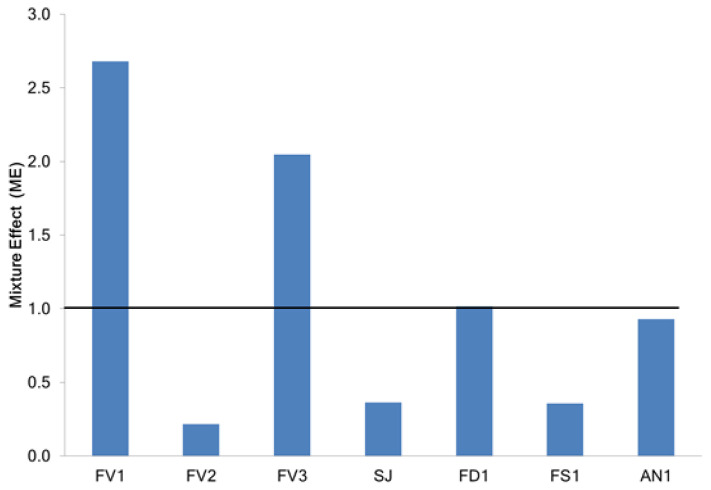
The mixture effect (ME) of fucoidans extracted from the brown seaweeds. *F. vesiculosus* (FV1, FV2, FV3), *S. japonica* (SJ), *F. distichus* (FD1), *F. serratus* (FS1), and *A. nodosum* (AN1).

In this study, for the assessment of ME, solutions of the fucose (main structural unit of fucoidan) and phloroglucinol (as representative of polyphenols) were prepared, and their ability to scavenge DPPH radicals were studied. Based on the data on the DPPH-scavenging activity of individual compounds and fucoidans, the ME was calculated (Figure 6).

Phloroglucinol was very effective in scavenging DPPH free radicals with an IC_50_ of 5.6 ± 0.5 μg/mL. At the same time, fucose showed a very low effect in this assay (IC_50_ 310 ± 9 μg/mL). The data obtained are consistent with those previously reported [36,37]. In the present study, most of the fucoidans tested, except FV2, were found to contain high concentrations of phenolic compounds (Table 1). It was the FV2 sample that had a pronounced antagonistic effect (ME 0.21), while other samples of fucoidan from *F. vesiculosus* (FV1 and FV3) with an increased TPC showed a synergistic effect (ME 2.68 and 2.04, respectively). Fucoidans from *F. distichus* and *A. nodosum*, despite the significant number of total polyphenols (72.8 mg/kg and 62.4 mg/kg, respectively), did not show a synergistic or antagonistic effect (ME 1.02 and 0.93, respectively). Fucoidans from *S. japonica* and *F. serratus* showed an antagonistic effect (ME 0.36 and 0.35, respectively) (Figure 6). A strong positive relation between ME and total polyphenols (Pearson’s correlation coefficient r = 0.933, *p* < 0.05) and a moderate positive relationship between ME and fucose content (Pearson’s correlation coefficient r = 0.436, *p* < 0.05) were found. Likely mono sugars, without exerting individual antiradical action, stabilize fucoidan polyphenols and possibly participate in the oxidation reaction as a reducing agent.

Our results support the substantial role of carbohydrates in synergy with phenolics for antioxidant activity. According to the total antioxidant capacity test, fractions of sugars and organic acids were both antagonists and synergists in combinations with their polyphenols in fruit juices [38]. Glucose, sucrose, and fructose solution contributed to the synergy between rutin and *p*-coumaric acid in the ORAC assay [39]. N. Belaya et al. (2019) reported a synergistic effect of binary mixtures of quercetin: monosaccharide in the model reaction with DPPH radical in deoxygenated ethanol. It was shown that the studied tetroses, pentoses, and hexoses showed a synergistic effect, enhancing the antiradical effect of quercetin. According to other authors, the synergistic effect of the mixture depends on the number of hydroxyl substituents and the presence of aldehyde or ketone groups in carbohydrate molecules [37]. The binary mixtures of polyphenolic compounds with high molecular weight pectin (containing 200–1000 units of galacturonic acid with different degrees of esterification) showed additive or antagonistic effects in DPPH and FRAP assays. The antioxidant capacity depended on the type and the location of hydroxyl and the type of phenolic compounds [40].

#### 2.2.3. Inhibition of Proteins Denaturation and Membrane Stabilizing Effect

**Figure 7:**
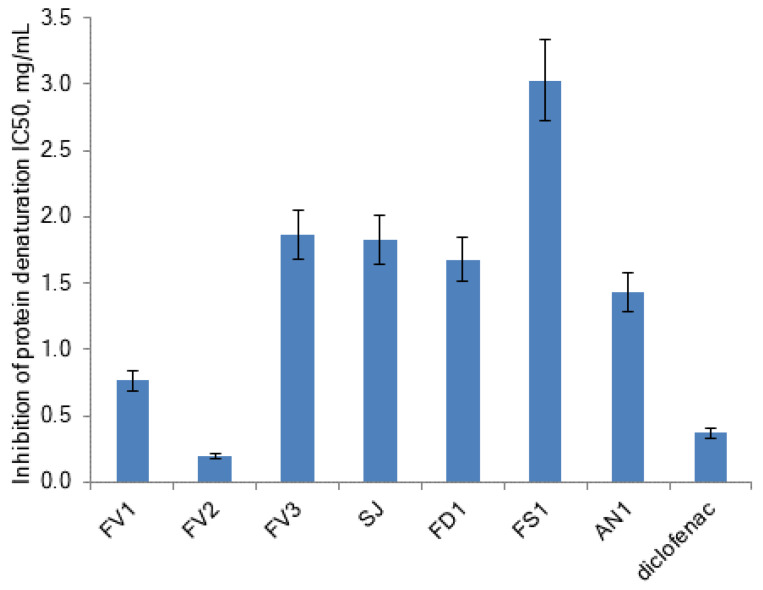
Effects of fucoidans extracted from brown seaweeds on protein denaturation. *F. vesiculosus* (FV1, FV2, FV3), *S. japonica* (SJ), *F. distichus* (FD1), *F. serratus* (FS1), and *A. nodosum* (AN1).

Inflammation is associated with pain and temperature escalation and leads to protein denaturation and an increase in vascular membrane alteration and permeability [41]. Protein denaturation has a complicated mechanism that includes the modification of electrostatic hydrogen, hydrophobic, and disulfide bonds [42]. In inhibition of the protein denaturation method, egg albumin denaturation was induced by heat. Effects of fucoidans extracted from brown seaweeds on protein denaturation are presented in Figure 7.

**Figure 8:**
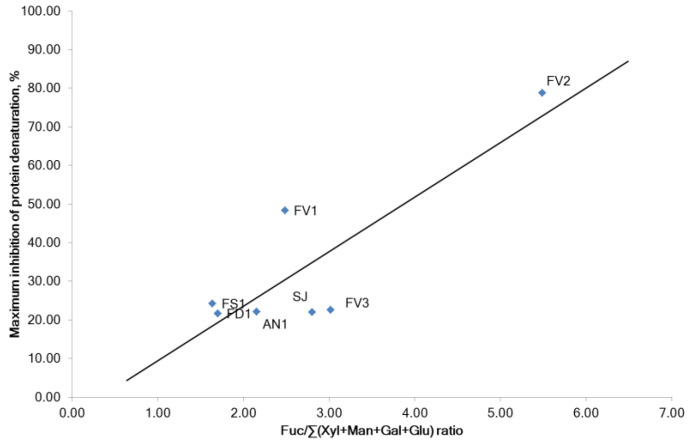
Effects of sugars proportion in fucoidans from brown seaweeds on inhibition of protein denaturation. *F. vesiculosus* (FV1, FV2, FV3), *S. japonica* (SJ), *F. distichus* (FD1), *F. serratus* (FS1), and *A. nodosum* (AN1).

The protein (albumin) denaturation was concentration-dependently inhibited by fucoidans and diclofenac sodium at the concentration range of 0.05–0.75 mg/mL. A strong correlation was found between the anti-inflammatory effect (inhibition of protein denaturation) and the content of carbohydrates and molecular weight of fucoidan (Pearson’s correlation coefficients r = 0.848 and −0.744, respectively, *p* < 0.05). The correlation of inhibition of protein denaturation with the content of sulfates was moderate (Pearson’s correlation coefficient r = 0.478, *p* < 0.05). Surprisingly, purified fucoidan FV2 was significantly powerful inhibitor of proteins denaturation (IC_50_ = 0.20 mg/mL) than diclofenac sodium (IC_50_ = 0.37 mg/mL). It was noted that the inhibition of protein denaturation was increased with the increase in the relative fucose content in carbohydrates ((Fuc/Σ(Xyl + Man + Ga + Glu) ratio) (Figure 8). The correlation between the anti-inflammatory activity of fucoidans and TPC was extremely weak (Pearson’s correlation coefficients r = −0.211, *p* < 0.05).

Various non-steroidal anti-inflammatory drugs (NSAID) have previously been shown to inhibit the denaturation of biologically active plasma proteins in vitro [43]. Denaturation affects nearly all physicochemical properties of protein molecules [44]. Protein denaturation may be the initial step in further protein modification, such as protein glycosylation. Phenomena of abnormal protein glycosylation often occur in chronic inflammatory diseases [45]. The implementation of the erythrocyte membrane stabilization model for anti-inflammatory screening is based on the structural similarity between erythrocyte membranes and lysosomes. Lysosomes are significant organelles that induce the mechanism of action in the late inflammatory response by releasing bactericidal enzymes and various proteases. Thus, maintaining their structural integrity is critical for suppressing the inflammatory process [46]. Due to the similarity of the lysosomal membrane to the erythrocyte membrane, the model of stabilization of human red blood corpuscles (HRBC) membrane has been used for the estimation of the anti-inflammatory activity of plant extracts. [47].

**Figure 9:**
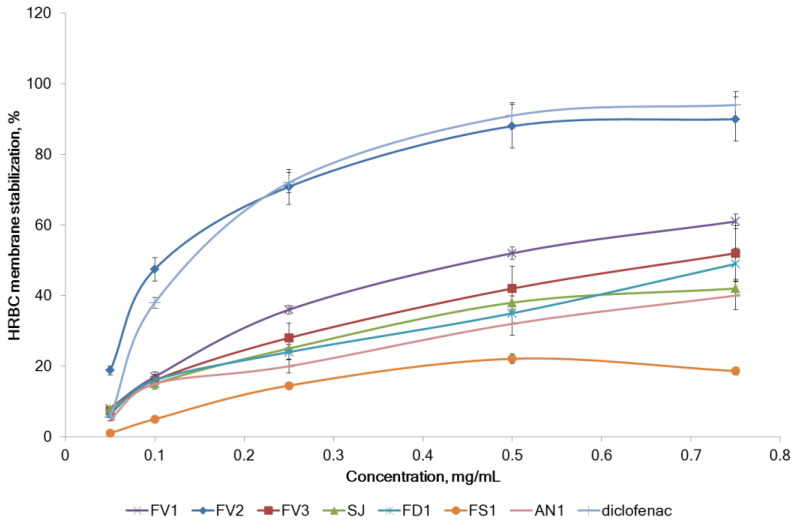
Effects of fucoidans extracted from the brown seaweeds on HRBC membrane stability. *F. vesiculosus* (FV1, FV2, FV3), *S. japonica* (SJ), *F. distichus* (FD1), *F. serratus* (FS1), and *A. nodosum* (AN1).

The HRBC membrane stabilization by fucoidan from various brown seaweeds is shown in Figure 9. The samples had different effects on the stabilizing activity of the membranes. The FV2 sample was comparable to diclofenac sodium and had the highest membrane stabilizing ability compared to other samples. A 0.5 mg/mL of FV2 and diclofenac showed 88% and 91% protection, respectively. FV3, FD1, and SJ had a sufficiently high membrane stabilizing activity compared to other samples. A 0.75 mg/mL FV3 showed 52% protection. The stabilization of HRBC membranes by fucoidans was in the same shape as the inhibition of proteins denaturation IC_50_ (Pearson’s correlation coefficient r = −0.930, *p* < 0.05).

It was reported that seaweed polysaccharides showed significant anti-inflammatory activities in vivo and in vitro [4,48,49]. The purified fucoidan fraction from *Turbinaria ornate* (38% sulfates, 0.25% TPC, and 60.4% carbohydrates, among which 86% fucose) reduced ROS and NO expression in zebrafish embryos [14], reduced an arthritic score, and paw volume of rats with Freund’s Adjuvant induced arthritis [13]. Similar anti-inflammatory effects were observed in rats after per-oral administration of commercial fucoidan with high fucose content from *Undaria pinnatifida* [50]. The cream with fucoidan from *F. vesiculosus* (MW 735 kDa; 27% sulfate and 79.5% carbohydrates, among which 75.3% fucose) inhibited carrageenan-induced edema and ameliorated mechanical allodynia in rats after topical application [19].

The anti-inflammatory potential of fucoidan was mediated via different mechanisms. Purified fucoidan fraction from *T. ornate* down-regulated iNOS, COX-2, and pro-inflammatory cytokines, including PGE2 levels in RAW 264.7 macrophages [14]. Purified fucoidan from *Ecklonia maxima* (fucose content 81%) reduced prostaglandin E2, NO, and pro-inflammatory cytokines, such as TNF-α, IL-6, and IL-1β in macrophages [18]. A fucoidan from brown algae *A. nodosum* inhibited poly(I:C)-induced expression of some cytokines [51]. The fucoidan from *F. vesiculosus* (MW 735 kDa; 27% sulfate and 79.5% carbohydrates, among which 75.3% fucose) showed remarkable anti-inflammatory activity through inhibition of COX-1/2, hyaluronidase, and MAPK p38 [15].

In this study, new mechanisms of anti-inflammatory activity by (i) inhibition of egg protein denaturation and (ii) stabilization of HRBC membranes were established for fucoidan from five brown seaweeds. The results of our experiments are in line with the previously published. Fucoidan from *Sargassum wightii* (27.5% of sulfate and 31.8% of fucose prevented the protein denaturation effect on bovine albumin solution (BSA) [52]. Sulfated polysaccharide from *S. swartzii* (MW 1840 kDa, fucose is the main sugar) showed inhibition of BSA denaturation by 49.1% at a concentration of 10 μg/mL [53]. It was reported that auto-antigen production in certain inflammatory diseases could be associated with the denaturation of proteins [42]. The results of our study suggest that fucoidans can inhibit protein denaturation and are possibly capable of modifying the synthesis of autoantigens. The crude extract from Turbinaria ornate (rich in carbohydrates and phenolics) was reported to protect erythrocyte hemolysis induced by H_2_O_2_ in the human RBC model by maintaining the cell membrane integrity [54]. Fucoidan from *S. wightii* (100 μg/mL) protected membranes of HRBC for 80.5% [52].

All the above-mentioned literature data and results of the current study suggest the importance of high sulfation and fucose content for the anti-inflammatory activity of fucoidans, while this activity was not closely related to polyphenols content. Purification and fractionation of fucoidans lead to an increase in the relative content of carbohydrates and fucose (as the main carbohydrate). This may play an important role in anti-inflammatory effects. However, future investigations are required for confirmation of this hypothesis.

## 3. Materials and Methods

### 3.1. Materials

Brown seaweeds, bladder wrack (*Fucus vesiculosus* L.), toothed wrack (*Fucus serratus* L.), rockweed (*Fucus distichus* L.), and kelp Icelandic (*Ascophyllum nodosum* L.) Le Jolis) were from the littoral of the Barents Sea (bay Zelentskaya, Murmansk region, Russia) in August–September 2021. The seaweeds were identified by Dr. E. Obluchinskaya, and the voucher specimens (No. 9.2021, V.D.Z.) were deposited in the Collection of the Zoobentos Laboratory, Murmansk Marine Biology Institute. Collected seaweeds were thoroughly washed in running tap water followed by distilled water to remove the adhering sand, epiphytes, extraneous matter, and necrotic parts. The samples were frozen and stored at −25 °C for later extraction. The Folin–Ciocalteu reagent, ascorbic acid, phloroglucinol, and 2,2-diphenyl-1-picrylhydrazyl (DPPH), ammonium molybdate were from Sigma-Aldrich (St. Louis, MO, USA) and diclofenac sodium was from Calbiochem (San Diego, CA, USA). Fucoidan from *Saccharina japonica* was provided by Changsha Vigorous-Tech Co., Ltd. (Hunan, China). Polychrome-1 (fluorine-containing polymer from Teflon brand 4D, fraction 0.20–0.50 mm) was from Reahim LLC (Moscow, Russia). The water was purified via a Milli-Q system (Millipore, Bedford, MA, USA). Other analytical grade chemicals and solvents for extraction and assay were of analytical grade and purchased from local chemical suppliers.

### 3.2. Extraction Procedures

Method I. The extraction of fucoidan reported previously [21] with some modifications was used. Briefly, frozen seaweed samples (100 g) were ground and pretreated with chloroform, acetone, and EtOH. Samples of defatted, dry, and powdered alga fronds were extracted in an ultrasound bath (Branson 3510DTH, Branson Ultrasonics Corp., Danbury, CT, USA) with a 5% aq. EtOH at 40 °C for 4 h. The collected supernatant was concentrated ultrafiltration using a column filled with hollow fibers and simultaneously dialyzed by adding purified water and freeze-dried (samples of fucoidan from *F. vesiculosus* (FV1), *F. serratus* (FS1), *F. distichus* (FD1), and *A. nodosum* (AN1), respectively). 

Method II. Additionally, the sample FV1 was subjected to hydrophobic chromatography on Polychrome-1 [22]. The fractions were eluted with water, 5% aq. EtOH, and 15% aq. EtOH successively until the eluates were free from carbohydrates with phenol–sulfuric acid reagents. Glacial HOAc was slowly added to the water-eluted fractions (approximately up to 40%) to precipitate the fractions of water-soluble fragments of alginic acid [55]. The fucoidan (FV2) was precipitated from the supernatant with aq EtOH (80%) (sample of fucoidan from *F. vesiculosus* FV2). 

Method III. A sample of fucoidan from *F. vesiculosus* FV3 was extracted by method II as reported [23]. Briefly, frozen seaweed biomass (100 g) 100 was ground and pretreated with 0.5 L of a methanol–chloroform–water (4:2:1) mixture at room temperature, the supernatant was centrifuged, and the algae residue was treated twice with the same mixture of solvents. The algae residue was extracted with 1 L of 2% CaCI_2_ aqueous solution at room temperature. The extract was separated by centrifugation, the residue was re-treated under the same conditions, and then twice extractions were performed at 70 °C. The combined extract was dialyzed against distilled water and concentrated to a volume of 0.2 L freeze-dried (sample of fucoidan from *F. vesiculosus* FV3).

The dried fucoidans were packed in an airtight until further use. 

### 3.3. Analysis of Fucoidan Composition

The molecular weight of fucoidan was analyzed according to V. Golovchenko et al. [56]. The aqueous solution of fucoidan was separated over Shodex Asahipak (Kanagawa, Japan) GS-620 HQ column (7.6 × 300 mm) and a GS-26 7B precolumn (7.6 × 50 mm). Standard pullulans with MWs 6.2–740 kDa (Polymer Laboratories, Houston, TX, USA) were used for the calibration of columns. The phenol-sulfuric acid method was applied for the analysis of total neutral carbohydrate (TNC) content in fucoidan [57]. The sulfate residues were determined by the BaCl_2_-gelatin method [58]. The monosaccharides were analyzed by a high-performance liquid chromatography system (HPLC Model LC 20 AT Prominence, Shimadzu, Kyoto, Japan) fitted with a Refractive index detector (RID-10A, Shimadzu, Japan) as described previously [59]. The concentration of monosaccharides was determined by hydrolysis of the fucoidan samples (10–15 mg) with 2 M trifluoroacetic acid (0.5 mL) at 121 °C for 2 h. Hydrolyzed polysaccharides were cooled in an ice water bath, centrifuged at 5000 rpm for 5 min, and the liquid fraction was neutralized to pH 7 with 2 M NaOH. Samples were injected into a Shodex Asahipak NH2P-50 4E (4.6 × mm, 5 µm) (Kanagawa, Japan) column at 50 °C in isocratic conditions with a mobile phase composed of 0.25 M H_3_PO_4_—acetonitrile (20:80, *v*/*v*) at a flow rate of 1.0 mL min^−1^. Typical HPLC chromatogram of the reference monosaccharides and typical HPLC chromatogram of sample FV1 are presented in Supplementary Materials Figures S1 and S2. Monosaccharides of analytical grade (fucose (Fuc), xylose (Xyl), mannose (Man), galactose (Gal), arabinose (Ara), and glucose (Glc)) (all contents >99%) were purchased from Sigma Aldrich (St. Louis, MO, USA). The Folin–Ciocalteau method was used for the analysis of total polyphenols content (TPC) [60]. The results are expressed as milligrams of phloroglucinol equivalent per gram (PhE/g) of fucoidan.

### 3.4. Determination of Antiradical/Antioxidant Activity

The DPPH scavenging activity was analyzed according to W. Brand-Williams et al. [61] with some modifications [25] by a spectrophotometer Shimadzu UV 1800 (Shimadzu, Kyoto, Japan). The DPPH radical scavenging activities were calculated using the following Equation (1):

(1) $$\mathrm{DPPH radical scavenging}\;\mathrm{(\%)}=(\frac{A_{c}-A_{s}}{A_{c}})\times 100\%$$

where $A_{c}$ is the absorbance of the control, and $A_{s}$ is the absorbance of the sample.

The IC_50_ represents the concentration of the tested sample or standard antioxidant required to scavenge 50% of the DPPH-radical. The antiradical power (ARP) was calculated as ARP = 1/IC_50_ [25].

The total antioxidant capacity (TAC) of samples of fucoidan was evaluated as described [28]. Briefly, 0.1 mL of the aqueous solution of fucoidan (50–750 µg/mL) was combined in an Eppendorf tube with 1 mL of reagent (0.6 M sulfuric acid, 28 mM sodium phosphate, and 4 mM ammonium molybdate). The tubes were incubated at 95 °C for 90 min. After cooling of samples, the absorbance was recorded on Shimadzu UV 1800 spectrophotometer at 695 nm against a blank. A blank solution contained 1 mL of reagent solution and the appropriate volume of water. TAC was expressed as mg ascorbic acid equivalents/mg sample (AscAE/mg).

### 3.5. Determination of Mixture Effect

The mixture effect (ME) was calculated according to [33] to Equation (2):

(2) $$ME=\frac{{ARP}_{exp}}{{ARP}_{cale}}$$

where *ARP_exp_* is the experimental ARP of the extract and *ARP_calc_* is the expected *ARP*, calculated by the sum of efficiencies of each compound individually.

### 3.6. Inhibition of Proteins Denaturation

The protein denaturation method described by P. Padmanabhan et al. [12,62,63] was adopted and used to evaluate the anti-inflammatory activity of the fucoidan samples. Briefly, the reaction mixture containing 2 mL of fucoidan aqueous solution (50–750 µg/mL) or reference drug diclofenac sodium (50–750 µg/mL) and 2.8 mL of phosphate-buffered saline (pH 6.4) was mixed with 2 mL of egg albumin (from fresh hen’s egg) and incubated at (27 ± 1) °C for 15 min. Denaturation was induced by keeping the reaction mixture in a water bath at 70 °C for 10 min. After cooling, the absorbance was measured at 660 nm by Shimadzu UV 1800 (Shimadzu, Kyoto, Japan) spectrophotometer. The increase in absorbance by fucoidan/reference drug samples (*A_s_*) compared with control (*A_c_*) indicated stabilization of protein, i.e., inhibition of heat-induced albumin denaturation. The percentage inhibition of protein denaturation was calculated (Equation (3)):

(3) $$\mathrm{Inhibition}\;\mathrm{(\%)}=(\frac{A_{s}-A_{c}}{A_{c}})\times 100\%$$

### 3.7. HRBC Membrane Stabilization

HRBC suspension was prepared according to Shafay et al. [64]. The blood was obtained from a healthy human volunteer who did not take NSAIDs for two weeks before the experiment and was enrolled in the study after signing informed consent. The procedure was approved by the Institutional Review of Murmansk Marine Biological Institute (6732/253-ok, 1 December 2016) and was performed in accordance with the Helsinki international ethical standards on human experimentation. Venous blood was collected into EDTA-coated tubes (1.6 mg/mL). RBCs were isolated from the whole blood by washing (centrifugation, 380× g, 10 min) 3 times with an equal volume of normal saline. The blood volume was calculated and reconstituted as 10% *v*/*v* suspension with normal saline (10% stock solutions were performed by mixing 1 mL RBC suspension with 9 mL distilled water).

The HRBC membrane-stabilizing method in the hypotonic solution described in [65,66] was used to detect the in vitro anti-inflammatory activity of the fucoidan samples. Briefly, the reaction mixture containing 0.5 mL of fucoidan aqueous solution (50–750 µg/mL) or reference drug diclofenac sodium (50–750 µg/mL) and control (distilled water instead of hyposaline to produce 100% hemolysis), 1 mL of phosphate buffer (pH 7.4), 2 mL of hyposaline (0.36% sodium chloride), and 0.5 mL of 10% RBCs suspension was incubated at (37 ± 1) °C for 30 min and then centrifuged at 380× g for 10 min. The supernatant absorbance (*A_s_*) was measured at 560 nm using spectrophotometer (UV 1800, Shimadzu, Kyoto, Japan). The percentage of HRBC membrane stabilization protection was calculated by Equation 4:

*HRBC membrane stabilization protection* (%) = 100 × (*A_c_* − *A_s_*)/*A_c_* (4)

### 3.8. Statistical Analysis

All experiments were performed in triplicate (*n* = 3). Data are expressed as mean ± standard deviation (±SD), and error bars in the figures indicate standard deviation. Statistical analysis was performed with STATGRAPHICS Centurion XV (StatPoint Technologies Inc., Warrenton, VA, USA). Differences between means were analyzed by the ANOVA test followed by the post hoc Tukey’s test. A significant difference was considered at the level of *p* < 0.05. Pearson’s correlation coefficients were used to establish the relationship between the content of representative compounds and antiradical activity. Multiple regression and multivariate data analysis as partial least squares coefficient method were carried out.

## 4. Conclusions

In this study, fucoidans from five brown seaweeds were obtained and characterized. The anti-inflammatory activity of fucoidans was tested in vitro. The ARP of fucoidans strongly and positively correlated with TPC. A weak correlation was associated with xylose content. Similar effects were observed in the TAC test, where the activity of fucoidan was dose-dependent. Notable, the TAC of fucoidans except samples of FV2 and SJ (which have a lower polyphenol content) was higher than that of phloroglucinol. The synergistic effect of carbohydrates and polyphenolic compounds was calculated for the first time for the radical scavenging activity of fucoidan from five brown seaweeds. Only fucoidans from *F. vesiculosus* (FV1 and FV3) showed synergy with ME > 1. A strong positive relation between TPC and ME was noted. The correlation between ME and fucose content was moderately positive. Presumably, monosaccharides, without exerting individual antiradical action, stabilize fucoidan polyphenols and possibly participate in the oxidation reaction as a reducing agent.

Using the model of the denaturation of egg albumin, it was found for the first time that the anti-inflammatory activity of fucoidan could be mediated via inhibition of proteins denaturation. The inhibition was concentration-dependent and was increased with the increase in the fucose content in fucoidan and moderately correlated with sulfates. The correlation with molecular weight was negative. Purified fucoidan FV2 proved to be the most potent inhibitor of protein denaturation and surpassed diclofenac sodium in activity. Similar relations were also observed in the membrane protection model. Fucoidans were able to stabilize the cell membrane integrity of human RBCs. 

Taking into account the complex nature of inflammation, its therapy requires the application of multitarget drugs with different mechanisms of activity. The results of our study support the rationality of fucoidan use as a promising antiradical agent, inhibitor of protein denaturation, and RBC membrane stabilizer for the treatment of inflammatory-related diseases.

## Figures and Tables

**Table 1:** Composition of the fucoidans of the brown seaweeds.

Seaweed Source	Sample Code	Method of Extraction	Yield (% DW)	Total Neutral Carbohydrate (g/100 g DW)	Sulfate (g/100 g DW)	Total Polyphenols Content (PhE/g DW)
*Saccharina japonica*	SJ	-	-	48.4 ± 2.5	28.1 ± 0.2	10.1 ± 0.1
*F. vesiculosus*	FV1	I	22.3	64.1 ± 0.6	25.9 ± 2.6	139.6 ± 0.7
*F. vesiculosus*	FV2	II	6.6	62.6 ± 0.4	25.4 ± 1.0	13.3 ± 0.3
*F. vesiculosus*	FV3	III	8.2	53.7 ± 0.6	20.9 ± 1.5	132.2 ± 0.7
*F. distichus*	FD1	I	15.4	50.7 ± 1.1	17.6 ± 0.3	72.8 ± 0.6
*F. serratus*	FS1	I	13.6	45.5 ± 1.5	15.1 ± 0.1	64.0 ± 0.4
*A. nodosum*	AN1	I	16.1	49.9 ± 0.1	19.2 ± 0.1	62.4 ± 0.2

Each value represents mean ± SD of three determinants.

## Data Availability

Data is contained within the article or Supplementary Material.

## Supplementary Materials

The following supporting information can be downloaded at: https://www.mdpi.com/article/10.3390/md20100606/s1, Figure S1: Typical HPLC chromatogram of the reference monosaccharides; Figure S2: Typical HPLC chromatogram of sample FV1.
