# Supplementary material for: In Vitro Anti-Inflammatory Activities of Fucoidans from Five Species of Brown Seaweeds
Source: Mar Drugs. 2022 Sep 27;20(10):606. doi: 10.3390/md20100606 (PMC9605532; doi:10.3390/md20100606)
Supplement: Supplementary file 1 [file marinedrugs-20-00606-s001.zip › marinedrugs-1898879-supplementary.pdf]

Supplementary materials

In-Vitro Anti-Inflammatory Activities of Fucoidans from Five Species of Brown Seaweeds

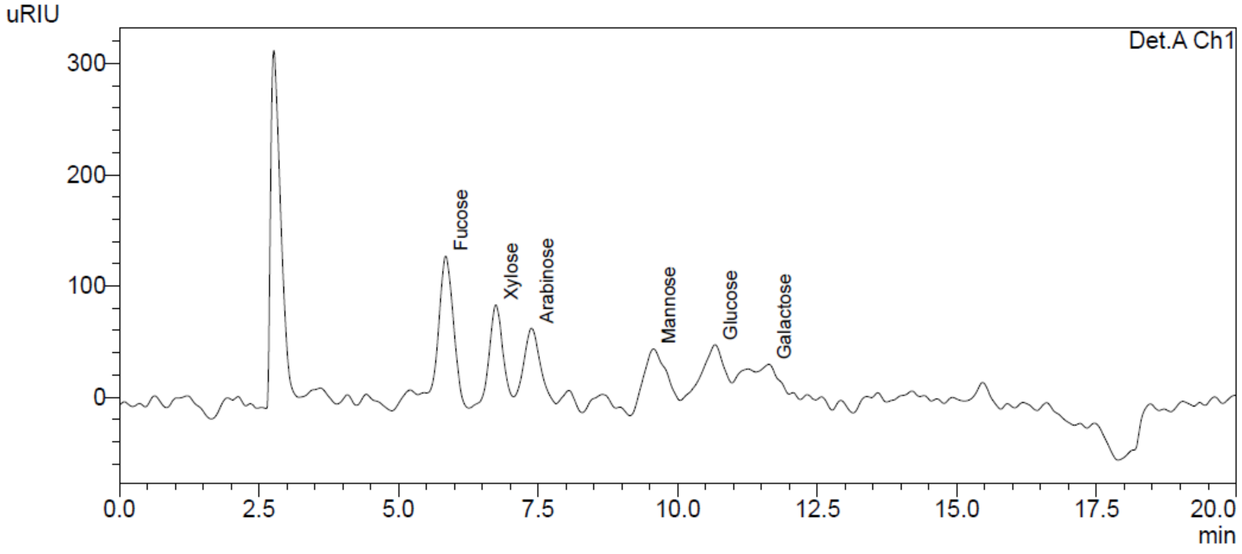

Figure S1. Typical HPLC chromatogram of the reference monosaccharides

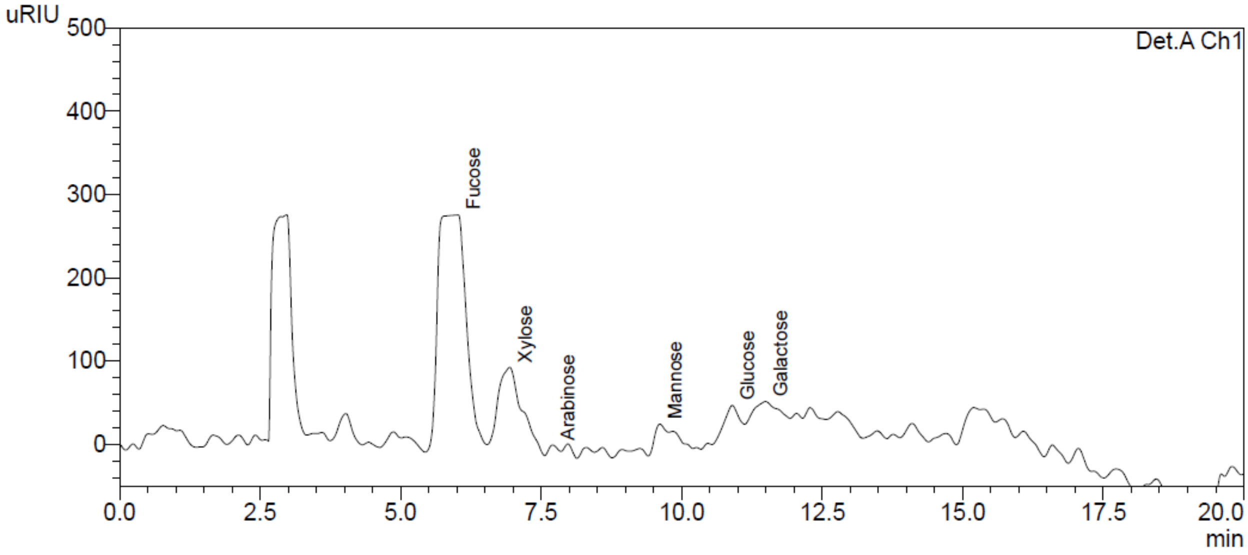

Figure S2. Typical HPLC chromatogram of sample FV1
